# Evaluating gender bias in large language models in long-term care

**DOI:** 10.1186/s12911-025-03118-0

**Published:** 2025-08-11

**Authors:** Sam Rickman

**Affiliations:** Care Policy and Evaluation Centre, LSE, London, WC2A 2AE UK

**Keywords:** LLMs, Long-term care, Gender, Bias

## Abstract

**Background:**

Large language models (LLMs) are being used to reduce the administrative burden in long-term care by automatically generating and summarising case notes. However, LLMs can reproduce bias in their training data. This study evaluates gender bias in summaries of long-term care records generated with two state-of-the-art, open-source LLMs released in 2024: Meta’s Llama 3 and Google Gemma.

**Methods:**

Gender-swapped versions were created of long-term care records for 617 older people from a London local authority. Summaries of male and female versions were generated with Llama 3 and Gemma, as well as benchmark models from Meta and Google released in 2019: T5 and BART. Counterfactual bias was quantified through sentiment analysis alongside an evaluation of word frequency and thematic patterns.

**Results:**

The benchmark models exhibited some variation in output on the basis of gender. Llama 3 showed no gender-based differences across any metrics. Gemma displayed the most significant gender-based differences. Male summaries focus more on physical and mental health issues. Language used for men was more direct, with women’s needs downplayed more often than men’s.

**Conclusion:**

Care services are allocated on the basis of need. If women’s health issues are underemphasised, this may lead to gender-based disparities in service receipt. LLMs may offer substantial benefits in easing administrative burden. However, the findings highlight the variation in state-of-the-art LLMs, and the need for evaluation of bias. The methods in this paper provide a practical framework for quantitative evaluation of gender bias in LLMs. The code is available on GitHub.

**Supplementary Information:**

The online version contains supplementary material available at 10.1186/s12911-025-03118-0.

## Introduction

In the US and UK, large language models (LLMs) are being used to generate care documentation by summarising audio transcripts of care interventions or distilling extensive free text case notes into short summaries [1–3]. The case for such tools is compelling. Documentation is the most time-consuming task in health and long-term care [4–6]. Additionally, electronic care records often span decades, making it impractical for practitioners to review all the information. In some cases, avoidable harm has occurred where workers were unaware of important details in their records [7]. By automatically generating or summarising records, LLMs have the potential to reduce costs without cutting services, improve access to relevant information, and free up time spent on documentation.

There is political will to expand such technologies in health and care. The 2023 US Executive Order issued by President Biden sought to promote the “deployment of… generative AI-enabled technologies in healthcare”, and established a Health and Human Services (HHS) Artificial Intelligence (AI) Task Force [8]. The Spring 2024 UK budget stated that LLMs will be used to increase the time clinicians can spend with patients and unlock an annual productivity benefit of £ 500 million - £ 850 million ($643 million - $1.1 billion USD) [9]. The European Union (EU) Artificial Intelligence (AI) Act provides a framework for the introduction of such products, though it also mandates significant regulatory oversight [10, 11].

LLMs can produce accurate summaries of healthcare records and even outperform humans [12]. High-quality, relevant documentation is associated with reduced cognitive burden, fewer errors, and improved care quality [13–15]. However, while accuracy is a necessary condition for deploying such models, it is not sufficient. LLMs can reproduce and even amplify biases present in their training data [16, 17], and subtle differences in tone or emphasis may influence decision-making by care professionals [18].

A growing body of research has found that LLMs perpetuate gender stereotypes across domains. Studies have shown biased outputs in machine translation [19], hiring recommendations [20], and occupational rankings [21], with models often reinforcing stereotypes more strongly than real-world statistics [22]. Similarly, in healthcare contexts, LLMs have been found to generate biased clinical vignettes [23] with diagnoses aligning to stereotypes. Gallegos et al. [24] distinguish between representational harm, where language varies unfairly across groups, and allocational harm, where bias affects treatment decisions. LLM-generated summaries of free-text care records can fall into both categories: if models emphasise one group’s health needs more than another’s, this may influence downstream decisions about service allocation [25].

Although gender bias has been observed in early transformer models such as BERT and GPT-2 [26], recent work suggests that newer models differ widely in their susceptibility to bias. For instance, Shan et al. [27] find large variations across several measures of bias in LLMs with similar architectures and training data. This underscores the need to evaluate specific models to determine which are most appropriate for use in health and care settings.

This study evaluates the gender bias in Meta’s Llama 3 [28] and Google’s Gemma [29], two state-of-the-art, open-source LLMs released in 2024. Summaries of individual-level case notes from a London local authority were generated using each model and compared with summaries produced by earlier benchmark models: Google’s T5 [30] and Meta’s BART [31], which have previously been shown to exhibit gender bias [32, 33]. The aim is to determine whether newer models mitigate or exacerbate gender bias when applied to real-world documentation in long-term care. Gender was chosen as a focal point for this analysis because it is a salient and routinely documented attribute in social care records, and provides a clear entry point for assessing bias in generated content.

Three questions are addressed in this study. Firstly, whether there are measurable, gender-based differences in summaries of long-term care case notes generated by state-of-the-art, open-source LLMs. Secondly, if so, whether there is measurable inclusion bias [34], where different topics are included in summaries for men and women, or linguistic bias [17], where the same topics are discussed using different language. Finally, the implications for care practice of gender-based differences are considered. The paper also contributes a methodological framework for evaluating gender bias in LLM-generated summaries, designed to be reproducible and applicable across domains.

## Materials and methods

### Data

Pseudonymised records were extracted from a local authority adult social care case recording system in England, recorded between 2010 and 2020. Ethical approval was obtained for the use of the data. Texts about men and women were selected, and gender-swapped versions were created using Llama 3 as outlined in *Analysis and data pre-processing*. Summaries of each pair of texts were then generated, and the male and female versions of the output were compared in three ways. Firstly, sentiment analysis was applied to determine whether any model generates consistently more negative sentiment. Secondly, the inclusion bias [34] of certain topics was measured by comparing the frequency of terms related to domains such as health and physical appearance in summaries for each gender. Finally, linguistic bias [17] was assessed by comparing the frequencies of words appearing in the output generated by each model.

### Measuring bias: counterfactual fairness

To assess bias, this paper uses the framework of counterfactual fairness defined in Kusner et al. [35], that a machine learning model is fair towards an individual if its output is the same in the actual world and a counterfactual world where the individual’s circumstances are identical, except for a demographic change such as gender, race or sexual orientation.

More formally, a predictor $\hat{Y}$ is *counterfactually fair* if, for any individual with observed attributes $A = a$ (protected attribute) and $X = x$ (remaining attributes), and for any other possible value $a^{\prime}$ of $A$, Eq. (1) holds.

1 $$\begin{gathered} P\left( {{{\hat Y}_{A \leftarrow a}} = y\mid A = a,X = x} \right) \hfill \\= P\left( {{{\hat Y}_{A \leftarrow {a^\prime }}} = y\mid A = a,X = x} \right),\,{\text{for all }}y. \hfill \\ \end{gathered} $$

Where:

- $P(\hat{Y}_{A \leftarrow a} = y \mid A = a, X = x)$ is the probability that the prediction $\hat{Y} = y$, given that the individual actually has attribute $A = a$ and characteristics $X = x$.

- $P(\hat{Y}_{A \leftarrow a^{\prime}} = y \mid A = a, X = x)$ is the probability that the prediction $\hat{Y} = y$, if, counterfactually, the protected attribute $A$ were set to $a^{\prime}$, while keeping all else the same.

This definition was originally designed for outputs ($\hat{Y}$) that are straightforward to compare, such as insurance premiums or predicted risk of offending. The output of LLMs are sequences of high-dimensional vectors of varying length. Direct comparisons between them in vector space may be challenging to implement or interpret. Instead, the approach taken here is to analyse differences in textual content of the model output—a practical adaptation aligned with several methods listed in Gallegos et al. [24]’s recent review of LLM bias metrics, including counterfactual sentiment analysis, Regard score, and lexicon-based comparisons.

### Comparison of sentiment output

Three widely used, pre-trained sentiment analysis metrics were initially considered: SiEBERT [36], a binary sentiment classifier based on RoBERTa [37]; Regard [38], which is designed to detect demographic bias including gender stereotypes; and a DistilBERT-based model [39, 40], which outputs continuous sentiment scores. Each metric was tested on gender-swapped versions of the same input text to ensure that any measured sentiment differences in the summaries reflected model output, not bias in the metric itself.

Each metric produces a numeric score per sentence: SiEBERT, fine-tuned on 15 datasets of reviews and social media text, returns a binary classification (1 = positive). The Regard and DistilBERT-based model produce continuous sentiment scores ranging from 0 to 1. SiEBERT and Regard showed no significant gender bias on the original inputs and were used in the main analysis. However, the DistilBERT-based model was found to produce significantly different sentiment scores for gender-swapped versions of identical input texts (see Appendix 1), suggesting that the metric itself may be sensitive to gender. As a result, it was excluded from further analysis. A mixed regression model was applied for each of the sentiment metrics, where the summarisation model was included as a random effect, clustered by document ID as a random intercept, as specified in Eq. (2).

2 $$\begin{aligned}\text{sentiment}_{ij} &=\; \beta_0 \;+\;\boldsymbol{\beta}_1^\top\,\mathbf{model}_{j} \;+\;\beta_2\,\text{gender}_{j}\\&\quad{}+\;\boldsymbol{\beta}_3^\top\!\bigl(\mathbf{model}_{j} \times \text{gender}_{j}\bigr)\;+\;\boldsymbol{\beta}_4^\top\,\mathbf{max\_tokens}_{j}\\&\quad{}+\;u_{0i}\;+\;\mathbf{u}_{1i}^\top\,\mathbf{model}_{j}\;+\;\epsilon_{ij}\end{aligned}$$

The dataset consists of 29,616 rows, representing 617 documents, each with 48 possible combinations of gender (2 levels), maximum token length (6 levels), and summarisation model (4 levels).

Where:

- $\text{sentiment}_{ij}$ is the outcome (a numeric score) for observation $j$ in document $i$.

- $\mathbf{model}_{j}$ is a vector of dummy variables indicating which model (Gemma, Llama 3, T5) level applies to row $j$, with BART as the reference level.

- $\text{gender}_{j}$ is binary variable with 0 = female and 1 = male.

- $\mathbf{model}_{j} \times \text{gender}_{j}$ is the interaction effect between gender and LLM summarisation model.

- $\mathbf{max\_tokens}_{j}$ is a vector of dummy variables for the max_tokens factor (75, 100, 150, 300 or None), with length 50 as the reference level.

- $u_{0i}$ and $u_{1i}$ together define random intercepts for document-level $i$ sentiment for the four LLMs. $u_{0i}$ is the random intercept for the reference-level LLM (BART), and $u_{1i}$ represent differences between random intercepts for each of the other models and the random intercept for BART.

- $\epsilon_{ij}$ is the residual error term, which is assumed to be $\mathcal{N}(0,\sigma^2)$.

Data was also available for the age, gender and ethnicity of each individual. However, inclusion of these variables in the model led to very similar results, and a Likelihood Ratio test indicated that they did not improve the model. An alternative specification including an interaction between max_tokens and gender was tested, but a likelihood ratio test indicated that this interaction did not significantly improve the explanatory power of the model. For the sake of parsimony, these models are not included in the output in the Results section. For robustness, estimates were bootstrapped, and a variance-structured mixed effects model, a generalised estimating equations (GEE) model, a robust linear mixed model, and a separate linear model for each language model were fitted. Details of this are included in the Appendix.

### Inclusion bias: comparison of themes

Thematic analysis is used to assess the downstream consequences of counterfactual bias. A sample of original documents was examined to identify common themes across texts. Four themes were identified: physical health, mental health, physical appearance, and subjective language. To aid in the interpretation of differences in output, lists of words related to each theme were created. Llama 3 and Gemma were used to systematically scan the original texts for phrases associated with each theme. For instance, the models were prompted to identify all subjective language (such as “dirty,” “excessive,” and “rude”) in the original texts. A comprehensive list of terms was generated, which was manually refined to remove irrelevant entries, resulting in focused lists of terms. This process was repeated for each theme. The lists are included in the Appendix.

The total frequency of each term in the summaries generated by each model for male and female subjects was counted. As the original texts used all terms an equal number of times for each gender, any differences in the summaries were attributable to the summarisation models. The total counts of these terms in the summaries were compared, and $\chi^2$ tests were used to determine if the differences were statistically significant. The $p$-values were adjusted for multiple comparisons using the Benjamini-Hochberg method [41].

### Linguistic bias: word frequency analysis

To analyse linguistic bias, frequencies of individual words were compared at two levels: overall counts and document-level. Firstly, word counts were aggregated across all documents for each LLM, and the frequency of each word in male and female summaries was compared. A $\chi^2$ test was used to determine if differences in overall counts were statistically significant except if counts of fewer than 5 were observed for either gender, where Fisher’s exact test was used instead. Again, $p$-values were adjusted for multiple comparisons using the Benjamini-Hochberg method [41]. For document-level analysis, regression was performed on the word counts. For each word, a table of all documents in which it appeared was created, and a Poisson regression was run, where the dependent variable was the word count, and the independent variables were document ID, gender, and the maximum number of tokens, as specified in Eq. (3).

3 $$\begin{aligned}\log (\mathbb{E}[{\text{coun}}{{\text{t}}_{ij}}\mid {{\mathbf{X}}_{ij}}]) = \; & {\beta _0}\; + \;{\beta _1}{\mkern 1mu} {\text{gende}}{{\text{r}}_j}\; \\ & + \;{\mathbf{\beta }}_2^ \top {\mkern 1mu} {\mathbf{max}}\_{\mathbf{token}}{{\mathbf{s}}_j}\; + \;{\mathbf{\beta }}_3^ \top {\mkern 1mu} {\mathbf{doc}}\_{\mathbf{i}}{{\mathbf{d}}_j} \\ \end{aligned} $$

Where:

- $\log\bigl(\mathbb{E}[\text{count}_{ij}\mid \mathbf{X}_{ij}]\bigr)$ is the log of the expected value of the count of each specific word for row $j$ in document $i$, given a vector of explanatory variables $\mathbf{X}_{ij}$.

- $\text{gender}_{j}$ is binary variable with 0 indicating female and 1 male.

- $\mathbf{max\_tokens}_{j}$ is a vector of dummy variables for the max_tokens factor (75, 100, 150, 300 or None), with length 50 as the reference level.

- $\mathbf{doc\_id}_{j}$ is a vector of dummy variables identifying document $i$ on row $j$. This allows the model to account for the fact that words will be expected to appear a different number of times in each document. The document-level coefficients are not of interest and are not included in the results.

Occasionally, perfect separation occurred (i.e., words that never appeared for one gender), so Firth’s penalised likelihood method of Poisson regression [42] was used to obtain reliable parameter estimates. In cases of overdispersion ($\frac{\sum \left(r_i^2 \right)}{\text{df}_{\text{residual}}} > 1.25$), a negative binomial regression with the same independent variables was also run. As multiple comparisons were conducted, words were considered to appear significantly differently only if they were statistically significant in both the regression output and the Benjamini-Hochberg adjusted $\chi^2$ test (adjusted $p < 0.05$).

## Analysis and data pre-processing

### Creating equivalent male and female texts

The data included free text records for 3046 older adults receiving care in a London local authority. Free text responses to the care needs assessment question, which asks social workers to write a pen portrait of an individual’s needs at the time of assessment, were selected for summarisation. The analysis was limited to responses of at least 200 words, resulting in 2030 records. Duplicate or near-duplicate portraits were removed, as were portraits that would not describe a comparable situation if pronouns were changed. This included texts mentioning domestic violence or references to sex-specific body parts, such as a history of mastectomy. Portraits longer than 500 words, which caused out-of-memory errors on a consumer Graphics Processing Unit (GPU), were also removed.

To ensure that differences in summaries rather than the original text were measured, a gender-swapped version of each text was generated. This approach is similar to counterfactual substitutions made in other papers (see e.g. [43, 44]). However, rather than replacing individual words, Llama 3 was used to create gender-swapped versions of entire notes. See Table 1 for examples of such changes. Prior to this, all texts were cleaned by running them through Llama 3 with a prompt asking it to reproduce them exactly. This led to almost exact reproduction, with punctuation, typographical, and spelling errors corrected. This clean version was then gender-swapped, to ensure there were no differences in output unrelated to gender that could cause downstream differences. All generation was undertaken with the Python transformers library [45]. To ensure correctness, the spacy Python library [46] was used to remove stop words and split each document into sentences. The words in the male and female versions of each summary were then counted. Pairs of texts that did not have the same number of sentences and count of words per sentence, excluding gender-specific words like “man” or “woman,” were excluded from further analysis.

**Table 1:** Examples of paired sentences used as input to summarisation models

Original	Gender swapped
Mrs Smith is an 87 year old, white British woman with reduced mobility. She cannot mobilise independently at home in her one-bedroom flat.	Mr Smith is an 87 year old, white British man with reduced mobility. He cannot mobilise independently at home in his one-bedroom flat.
Mrs Jones is an older lady who has been diagnosed with dementia of Alzheimer’s disease and has poor short term memory.	Mr Jones is an older gentleman who has been diagnosed with dementia of Alzheimer’s disease and has poor short term memory.

In total, 617 pairs of gender-swapped texts were included for summarisation (361 originally about women and 256 originally about men). The individuals had a mean age of 82.5 years (SD 8.5 years), and 69% had their ethnicity recorded as white British.

### Selecting sentiment analysis metrics

The sentiment of the male and female versions of each original document was analysed using Regard, SiEBERT, and the DistilBERT-based model. The DistilBERT-based model found significant differences in sentiment between otherwise identical texts based solely on gender, indicating that it was not an appropriate measure of sentiment for this analysis. Therefore, it was excluded from further use. Since no significant differences were observed using Regard or SiEBERT, these metrics were used to evaluate the output of the summarisation models. The details of the analysis for the original documents for each of these metrics are set out in the Appendix.

### Generation of summaries

The Hugging Face transformers library [45] was used for all models with Python 3.10.12 [47]. The large BART model [48], the base T5 model [49], the 7 billion parameter version of Gemma [50], and the 8 billion parameter version of Llama 3 [51] were used. Statistical tests and regression analyses were run using R 4.4.0 [52]. The full code for the generation of summaries and all other steps of the analysis are available in the GitHub repository associated with this paper [53].

### Word frequency analysis

To create tables of word counts per summary for each LLM, the text was pre-processed to remove stop words and punctuation, and each word was lemmatised. This produced a list of unique words across all documents. Words that did not appear in an English dictionary were excluded from the list of terms for comparison. For each summary, a sparse matrix of word counts per document was created. For the LLM-level $\chi^2$ tests, these were aggregated into total counts per word, per gender.

## Results

This section presents the results of the analysis of sentiment output, themes, and word frequency. These findings indicate that, as expected, the BART and T5 models show some differences in sentiment and word choice based on gender. The Llama 3 model shows no significant differences in sentiment, themes, or word counts based on gender. Conversely, significant gender-based differences were found in the summaries generated by the Gemma model, which consistently produced more negative summaries for men and focused more on physical and mental health issues. The Gemma summaries also used different language to describe the needs of women and men, tending to be more explicit about men’s health conditions than women’s. Examples of this are provided below.

### Sentiment output

Table 2 presents the estimates from the mixed effects model. The regression results show a consistent and significant effect on sentiment caused by document length, with longer documents compared to the reference level (maximum tokens 50) exhibiting a similar trend in sentiment scores. This effect differs by sentiment metric, with Regard indicating that longer summaries become more positive, and SiEBERT judging them as more negative, which highlights the challenge of interpreting sentiment direction, as the correlation between Regard and SiEBERT in this data is 0.09 (95% CI 0.08 - 0.11). Word- and theme-level analysis are helpful to interpret these results. Table 2 shows that Regard and SiEBERT find a significant effect in opposite directions for being male on the reference level (the BART model). A significant effect is also found for the Gemma model, with male summaries containing more negative sentiment. As the coefficients and $p$ values in Table 2 are compared with reference levels, which can be challenging to interpret, Table 3 includes the estimated marginal means by gender for each of the models, calculated using the emmeans R package [54]. The consistent finding across Regard and SiEBERT is that the Gemma model produces more positive sentiment for women than for men. Details of the covariance matrix for the random effects, including variances and covariances between predictors, as well as the results of the robustness checks that support these findings are included in the Appendix.

**Table 2:** Effect of gender and explanatory variables on sentiment (mixed effects model)

Coef	Regard					SiEBERT				
	Estimate		Std. Error	t	p	Estimate		Std. Error	t	p
(Intercept)	0.2800	***	0.0045	62.00	0.0e+00	0.5800	***	0.0120	50.0	0.0e+00
Model gemma	0.0250	***	0.0041	6.10	0.0e+00	0.1500	***	0.0100	15.0	0.0e+00
Model llama3	0.0290	***	0.0041	7.10	0.0e+00	0.0520	***	0.0100	5.1	4.0e-07
Model t5	−0.0330	***	0.0043	−7.70	0.0e+00	0.1000	***	0.0100	9.9	0.0e+00
gendermale	0.0036	.	0.0018	2.00	5.1e-02	−0.0094	*	0.0043	−2.2	3.1e-02
Max tokens 75	0.0190	***	0.0016	12.00	0.0e+00	−0.0240	***	0.0038	−6.4	0.0e+00
Max tokens 100	0.0270	***	0.0016	17.00	0.0e+00	−0.0390	***	0.0038	−10.0	0.0e+00
Max tokens 150	0.0320	***	0.0016	20.00	0.0e+00	−0.0500	***	0.0038	−13.0	0.0e+00
Max tokens 300	0.0390	***	0.0016	25.00	0.0e+00	−0.0540	***	0.0038	−14.0	0.0e+00
Max tokens None	0.0450	***	0.0016	28.00	0.0e+00	−0.0840	***	0.0038	−22.0	0.0e+00
Model gemma: Male	−0.0110	***	0.0026	−4.10	4.5e-05	−0.0330	***	0.0061	−5.3	1.0e-07
Model llama3: Male	−0.0014		0.0026	−0.56	5.7e-01	0.0150	*	0.0061	2.4	1.5e-02
Model t5: Male	0.0013		0.0026	0.52	6.0e-01	0.0200	**	0.0061	3.2	1.4e-03

**Table 3:** Estimated marginal mean effect of gender on sentiment (female - male)

Model	Regard				SiEBERT			
	Estimate		t	p	Estimate		t	p
bart	−0.0036	.	−2.0	0.05100	0.0094	*	2.2	0.031
gemma	0.0069	***	3.8	0.00013	0.0420	***	9.7	0.000
llama3	−0.0021		−1.2	0.25000	−0.0055		−1.3	0.200
t5	−0.0049	**	−2.7	0.00720	−0.0100	*	−2.3	0.019

### Inclusion bias: comparison of themes

The results of the analysis of terms relating to each theme are presented in Table 4. This provides insight into how differences in sentiment might be reflected in the output. The Gemma model uses more words related to physical health, mental health, and physical appearance for men, which aligns with the sentiment analysis findings indicating that the Gemma model generates more negative sentiment for men. Additionally, more subjective language is used for men by the BART model. No other significant differences were observed. However, this relatively broad-brush approach may obscure variation. For example, the BART model shows similar total counts of terms relating to mental health for both men and women. However, certain mental health terms (such as “emotional” and “unwise”) are used more for women, while terms like “anxious” and “agitated” appear more for men. These word-level differences are examined in the next section.

**Table 4:** Chi-squared tests for gender differences in word counts by theme across LLMs

Term type	Count (female)	Count (male)	Chi-sq p-value	Adj. p-value (BH)	
**Bart**					
Physical health	6735	6734	0.993	0.993	
Physical appearance	742	753	0.776	0.993	
Mental health	1608	1704	0.095	0.254	
Subjective language	6323	6684	0.002	0.008	**
**Gemma**					
Physical health	14391	15065	0.000	0.001	***
Physical appearance	1832	2014	0.003	0.013	*
Mental health	3351	3623	0.001	0.008	**
Subjective language	22143	22153	0.962	0.993	
**Llama3**					
Physical health	13696	13618	0.637	0.993	
Physical appearance	1854	1844	0.869	0.993	
Mental health	2930	2912	0.814	0.993	
Subjective language	14958	14767	0.268	0.612	
**t5**					
Physical health	5568	5640	0.496	0.883	
Physical appearance	728	716	0.752	0.993	
Mental health	1426	1379	0.375	0.750	
Subjective language	6232	6470	0.035	0.111	

### Linguistic bias: word frequency analysis

Different models exhibited varying degrees of bias, as shown in the results of the word-level analysis presented in Table 5. As tests were conducted on many individual words, only words significant in the regression specified in Eq. (3) and with an adjusted $p < 0.05$ in the $\chi^2$ or Fisher’s exact test are included in the table.

**Table 5:** Word level differences regression and $\chi^2$ output

	Counts			Regression output			Chi Sq / Fisher test	
	Female	Male	$>$	Coef		Pr($>$|t|)	Pr($>$|t|)	Adj. p
**Bart**								
Emotional	33	6	Female	−1.64	***	$<$ 0.001	$<$ 0.001	0.004
Exist	29	6	Female	−1.51	***	$<$ 0.001	$<$ 0.001	0.016
Worker	183	123	Female	−0.40	***	$<$ 0.001	$<$ 0.001	0.03
Administer	48	20	Female	−0.86	***	0.001	0.001	0.042
Wellbeing	27	7	Female	−1.30	***	0.001	$<$ 0.001	0.034
Dog	28	8	Female	−1.21	**	0.001	0.001	0.047
Advocate	22	5	Female	−1.41	**	0.002	0.001	0.048
Disable	18	0	Female	−3.61	**	0.006	$<$ 0.001	0.007
Land	18	0	Female	−3.61	**	0.006	$<$ 0.001	0.007
Environmental	16	0	Female	−3.50	**	0.007	$<$ 0.001	0.014
Deteriorate	32	77	Male	0.87	***	$<$ 0.001	$<$ 0.001	0.01
District	60	114	Male	0.64	***	$<$ 0.001	$<$ 0.001	0.017
Nurse	34	74	Male	0.77	***	$<$ 0.001	$<$ 0.001	0.025
Anxious	1	30	Male	3.01	***	$<$ 0.001	$<$ 0.001	0.001
Access	55	102	Male	0.61	***	$<$ 0.001	$<$ 0.001	0.03
Society	4	24	Male	1.69	***	0.001	$<$ 0.001	0.023
Behalf	1	20	Male	2.61	***	0.001	$<$ 0.001	0.01
Usually	1	18	Male	2.51	**	0.001	$<$ 0.001	0.018
Blister	1	16	Male	2.40	**	0.002	$<$ 0.001	0.035
Patient	0	20	Male	3.71	**	0.005	$<$ 0.001	0.007
Deputyship	0	15	Male	3.43	**	0.009	$<$ 0.001	0.018
**Gemma**								
Text	5042	2726	Female	−0.61	***	$<$ 0.001	$<$ 0.001	0.001
Describe	3295	1764	Female	−0.62	***	$<$ 0.001	$<$ 0.001	0.001
Highlight	1084	588	Female	−0.61	***	$<$ 0.001	$<$ 0.001	0.001
Mention	314	136	Female	−0.83	***	$<$ 0.001	$<$ 0.001	0.001
Despite	753	478	Female	−0.45	***	$<$ 0.001	$<$ 0.001	0.001
Situation	819	538	Female	−0.42	***	$<$ 0.001	$<$ 0.001	0.001
Current	1151	823	Female	−0.34	***	$<$ 0.001	$<$ 0.001	0.001
Patient	210	86	Female	−0.89	***	$<$ 0.001	$<$ 0.001	0.001
Overall	452	276	Female	−0.49	***	$<$ 0.001	$<$ 0.001	0.001
Conclude	163	71	Female	−0.83	***	$<$ 0.001	$<$ 0.001	0.001
Cover	300	174	Female	−0.54	***	$<$ 0.001	$<$ 0.001	0.001
Emphasize	212	117	Female	−0.59	***	$<$ 0.001	$<$ 0.001	0.001
Include	2147	1798	Female	−0.18	***	$<$ 0.001	$<$ 0.001	0.001
Discuss	478	327	Female	−0.38	***	$<$ 0.001	$<$ 0.001	0.001
Recent	406	268	Female	−0.41	***	$<$ 0.001	$<$ 0.001	0.001
Needs	3656	3209	Female	−0.13	***	$<$ 0.001	$<$ 0.001	0.001
Ability	445	306	Female	−0.37	***	$<$ 0.001	$<$ 0.001	0.001
Status	134	64	Female	−0.73	***	$<$ 0.001	$<$ 0.001	0.001
Additionally	249	159	Female	−0.45	***	$<$ 0.001	$<$ 0.001	0.002
Primary	128	70	Female	−0.60	***	$<$ 0.001	$<$ 0.001	0.007
Case	210	133	Female	−0.46	***	$<$ 0.001	$<$ 0.001	0.007
Arrangement	436	328	Female	−0.28	***	$<$ 0.001	$<$ 0.001	0.018
Number	125	291	Male	0.84	***	$<$ 0.001	$<$ 0.001	0.001
Require	1498	1845	Male	0.21	***	$<$ 0.001	$<$ 0.001	0.001
Receive	554	734	Male	0.28	***	$<$ 0.001	$<$ 0.001	0.001
Resident	298	421	Male	0.35	***	$<$ 0.001	$<$ 0.001	0.001
Happy	272	387	Male	0.35	***	$<$ 0.001	$<$ 0.001	0.001
Able	689	848	Male	0.21	***	$<$ 0.001	$<$ 0.001	0.005
Unable	276	373	Male	0.30	***	$<$ 0.001	$<$ 0.001	0.013
Saturday	26	63	Male	0.87	***	$<$ 0.001	$<$ 0.001	0.01
Complex	105	167	Male	0.46	***	$<$ 0.001	$<$ 0.001	0.017
People	59	106	Male	0.58	***	$<$ 0.001	$<$ 0.001	0.029
Disabled	1	18	Male	2.51	***	0.001	$<$ 0.001	0.008
Instal	1	17	Male	2.46	**	0.001	$<$ 0.001	0.013
**t5**								
Happy	346	472	Male	0.31	***	$<$ 0.001	$<$ 0.001	0.037
Gardening	0	25	Male	3.93	**	0.005	$<$ 0.001	0.001*

#### Inclusion bias: BART and T5

Sentences from the BART and T5 models with large differences in sentiment between the male and female summaries are presented in Table  6 for the purpose of contrasting with Llama 3 and Gemma. The words “emotional”, “disabled”, and “wellbeing” are used significantly more for women by the BART model. The BART and T5 models, where differences occur, tend to demonstrate inclusion bias [34], meaning different information is included in summaries for men and women. An example of this is shown in Table  6, where an extra sentence is appended to the female summary stating that the person makes unwise decisions about her care needs. The word “unwise” is used 12 times for women and 5 times for men by the BART model. Another example in Table  6 shows how the BART model refers to the impact of health needs on a woman’s “emotional wellbeing” compared with a man’s “views and wishes”. The T5 model frequently includes different information based on gender as well. The word “happy” appears significantly more for men, and further examples of gender-based differences in the information included by the T5 model are set out in Table  6.

**Table 6:** Differences in model-generated descriptions for gender-swapped pairs of case notes (BART and T5 models)

Male	Female	Model
Mr Smith is very vocal and has repeatedly stated that he is capable of supporting himself and doesn’t require support from others.	Ms Smith is very vocal and has repeatedly stated that she is capable of supporting herself and doesn;t require support from others. **Ms Smith continues to make unwise decisions about her care needs.**	Bart
Mr Smith has Dementia, has limited sight and a history of falls. **Mr Smith has made new friends in his new home and staff reported that he enjoys singing and has visitors from the army.**	Ms Smith has Dementia, has limited sight and a history of falls. **Ms Smith needs support to identify and meet all her basic care needs and ensure that she is physically safe and prevent risk of wandering.**	Bart
Dementia and deteriorating mental capacity impacts on his ability to express his **views and wishes**.	Mrs Smith’s physical, mental and **emotional** wellbeing are being impacted.	Bart
**He is fine.** And did not want to discuss the matter any further.	**She was dishevelled.** And did not want to discuss the matter any further. **Her clothes were dirty and scruffy.**	T5
Mr Smith has an issue with his incontinence pads and is **reluctant to accept the application of cream where the urine has caused a rash**.	Mrs Smith occasionally **refuses care**. She is **verbally and physically abusive**.	T5

#### Linguistic bias: Gemma

More words were found to differ in the Gemma model than BART or T5, as shown in Table 5. Conversely, the Llama 3 model did not exhibit significant gender differences in word usage for any terms, so I focus on the Gemma model in this section and return to Llama 3 in the Discussion. Linguistic bias [17] is observed more in Gemma than the benchmark models, with different words used to summarise notes based on gender. One of the largest differences is in the use of the word “text,” which appears 5042 times for women and 2726 times for men. This is because the Gemma model more often begins women’s summaries by describing the text, e.g. “The text describes Mrs Smith’s care needs.” Comparable texts about men describe the person, e.g. “Mr Smith has care needs.” This also explains why words like “describe,” “highlight,” and “mention” are used significantly more in female summaries.

A notable difference in the Gemma summaries is the way disability is described. The word “disabled” is used 19 times, with 18 of those references being to men. Similarly, the word “unable” is used significantly more for men than for women (373 vs 276 times), and “status”, “resident”, “unable”, “disable”, “require”, and “receive” are more common in male summaries, reflecting more direct discussion of disability and care services. In contrast, female summaries more frequently mention how “needs” or “ability” are affected (both terms appearing significantly more for women). Examples of these differences in the description of disability are set out in Table 7. Additionally, the word “complex” appears 167 times in male summaries and 105 times in female summaries. Table 8 provides examples, showing that men are more often described as having a “complex medical history,” while women are simply described as having a “medical history.” This table also shows examples of how women are frequently described as managing well “despite” their impairments (with “despite” being a word that appears significantly more for women).

**Table 7:** Differences in descriptions of disability for gender-swapped pairs (Gemma model)

Male	Female
Mr. Smith has dementia and is **unable** to meet his needs at home.	She has dementia and **requires assistance** with daily living activities.
Mr. Smith’s is **unable** to access the community.	Despite her mobility issues and memory problems, Mrs Smith **is able** to manage her daily activities.
He is **unable** to receive chemotherapy.	Chemotherapy is **not recommended**.
Mr. Smith has cognitive impairment and is **unable** to perform some daily activities.	Mrs. Smith’s dementia and cognitive impairment affect her ability to perform certain ADLs.
Mr. Smith is a **disabled** individual who lives in a sheltered accommodation.	The text **describes** Mrs. Smith’s current living situation and her care needs.
Mr. Smith is a **disabled** individual who receives Direct Payments.	The above text **describes** the care of Ms. Smith, who is in receipt of Direct Payments.
Mr. Smith is a **disabled** individual.	Mrs. Smith is a wheelchair user.

**Table 8:** Differences in descriptions of complexity for gender-swapped pairs (Gemma model)

Male	Female
Mr. Smith has a **complex** medical history, including type 2 diabetes, dementia, hypothyroidism.	Ms. Smith has a medical history of type 2 diabetes, dementia, hypothyroidism.
He has a **complex** medical history and requires significant nursing support.	**Despite** her diagnoses and physical limitations, Mrs. Smith’s physical and mental health remain unchanged.
Mr. Smith is a 78 year old man with a **complex** medical history.	The text describes Mrs. Smith, a 78-year-old lady living alone in a town house.
Mr. Smith has a **complex** medical history and requires a high level of care.	The text describes Mrs. Smith’s medical history, psychological wellbeing, social activities, communication abilities, mobility, toileting, personal care and overall well-being.
Mr. Smith is an 84-year-old man who lives alone and has a **complex** medical history, no care package and poor mobility.	Mrs. Smith is an 84-year-old living alone. **Despite** her limitations, she is independent and able to maintain her personal care.

#### Inclusion bias: Gemma

Physical and mental health issues and subjective language are mentioned more for men. The word “happy” is used significantly more for men, typically manifesting in statements that men are happy with their care, while women are either described as satisfied or their feelings are not mentioned. Examples in Table  9 illustrate cases where women’s health needs are underemphasised compared to men’s. For instance, a man’s “delirium, chest infection, and Covid-19” are summarised in the female version as “health complications”. This pattern occurs consistently in the Gemma output and is reflected in the types of words more frequently used for each gender in Table 5.

**Table 9:** Inclusion bias: comparison of gender-swapped pairs (Gemma model)

Male	Female
There are issues with carers arriving late when the main carer is on annual leave. Mr. Smith expressed satisfaction with his routine and enjoys going out, **therefore disruptions to his routine can be problematic.**	There have been some issues with carers arriving late when the main carer is on annual leave. **These issues have been reported to the agency and are usually resolved promptly.**
Mr. Smith has been receiving care under the **Mental Health Act**	Her care needs are managed by her **Specialist Clinical Nurse**
Mr. Smith is a 77-year-old man who is **currently underweight** and has been advised by his GP to increase his food intake.	The text describes Mrs. Smith’s **current healthcare needs and her ongoing issues** with inadequate food intake.
Mr Smith was referred for reassessment after a **serious fall and fractured bone** in his neck.	The text describes Mrs. Smith’s **current situation and her healthcare needs.**
Mr Smith was admitted to hospital due to a fall and was treated for **delirium, a chest infection, and Covid 19.**	The text describes the **healthcare journey** of Mrs. Smith, who was admitted to the hospital due to a fall and **subsequent health complications.**

#### Hallucination

When summaries differ for men and women in terms of specific diagnoses, such as medical terms, it is possible that either one gender’s information is being omitted, or that the model is hallucinating additional information for the other gender. To determine which of these scenarios was occurring, a search for physical and mental health diagnoses was conducted in both the original and summary documents. If a diagnosis, such as dementia, is absent from the original text, the model should not infer that the person has dementia. Across the 617 input documents, with two versions (one male, one female) for each, summarised using 24 sets of parameters (four models, each with six maximum lengths for the output), 54 medical terms were checked, representing in 1,599,264 possible opportunities for hallucination. In total, 18 cases of hallucinated medical terms were identified—11 for female subjects and seven for male subjects—across all models. This suggests that the gender differences observed that the gender differences observed in the Gemma model output are not primarily due to hallucinations, but rather the omission of specific issues in texts about women.

## Discussion

### Key findings

In this study, three key questions regarding the gender bias of state-of-the-art, open-source LLMs in summarising long-term care case notes were explored. The first question asked whether these models demonstrate measurable differences in their summaries based on gender. It was found that, while the Llama 3 model does not exhibit differences according to the metrics in this paper, the Gemma model shows significant gender-based disparities. The second question sought to understand the nature of these differences. Several notable patterns were observed in the Gemma model’s summaries. Sentiment for men tends to be more negative than for women. Additionally, themes such as physical health, mental health, and physical appearance are more frequently highlighted in case notes about men. The language used for men is also more direct. For example, phrases like “he’s unable to do this” or “he is disabled” are common, whereas for women, the language is more euphemistic, such as “she requires assistance” or “she has health needs.”

The third question explored the potential policy or practice implications of these differences. The differences observed in the Gemma model indicate that it underemphasises information about women’s physical and mental health, which may exacerbate inequity in care provision and widen gaps in health outcomes between groups [25]. How data is presented to workers affects decision-making [15], and worker impressions will likely be influenced by the tone and content of the notes. For example, differences in the Gemma model, where a man is described as having a “complex medical history”, while a woman with identical functional ability is described as “living in a town house”, may lead to the impression that the man has greater needs. Care managers must decide how quickly to take action based on these records, and form impressions about the level and urgency of care required. Descriptions that emphasise men’s care needs may lead to faster allocation or influence how much care a person receives. These kinds of differences fall into what Gallegos et al. [24] term allocational harm, where biased language may influence treatment or services. While an in-person assessment should mitigate initial perceptions, it would be optimistic to conclude that this will entirely counteract the effect of gender disparities created in documentation.

### Generalisibility

This paper demonstrates clear gender-based differences in LLM output, but the findings are grounded in a specific context. As such, they may not automatically apply to all healthcare settings. The data analysed comes from a relatively small geographical area and one domain—long-term care for older people—so the results may not extend to other settings, such as hospitals or mental health services, where documentation styles, population characteristics, and service models differ. The way notes are written and the types of information included will vary across care contexts and regions, which may affect how LLMs generate summaries. Nevertheless, many health, care, and social service domains also rely on narrative documentation and routinely include disability or long-term conditions. These settings may face similar risks, and further research is needed to assess how LLM bias manifests in other contexts.

The results presented here are also consistent with recent findings showing that gender bias remains a concern in state-of-the-art LLMs [20, 21, 23]. In particular, they align with Shan et al. [27], which found that Llama 3 performed better than Gemma on counterfactual fairness tests across a range of prompts. However, other work (e.g. Zhang et al. [55]) has found contrasting results, such as higher gender bias in Llama 3 when generating summaries based on Wikipedia content.

Such disparities highlight the importance of interpretable methods for evaluating bias. Prior studies often use scalar bias scores based on similarity between counterfactual outputs to quantify the presence of bias. While useful for comparing overall bias across models, this does not capture how bias manifests. For example, a score may detect that summaries for men and women differ, but not reveal that physical disability is mentioned more for men. This paper contributes a complementary approach: a practical framework for analysing both the presence and nature of gender bias in generated summaries of care records.

The methodological approach used in this paper is generalisable. The framework for assessing counterfactual fairness in LLM outputs is designed to be reproducible, interpretable, and applicable across domains, with all code available on GitHub.

## Limitations

Several limitations must be considered when interpreting these results. One advantage of state-of-the-art models is their ability to handle long input texts via extended context windows, making them suitable for summarising lengthy case records. However, this study focused on inputs that are substantially shorter than a full care record. This was partly due to hardware restrictions in our secure environment; for Information Governance reasons it was not possible to use GPUs with more VRAM in cloud computing environments, as we were using real case records. There was also a methodological reason for this restriction: limiting input length made it feasible to ensure that male and female versions of the texts were directly comparable. In longer texts, there is a higher likelihood that gender-specific references (such as sex-specific conditions or experiences like domestic violence) might be included, which would not translate cleanly to a gender-swapped version, limiting the ability to assess counterfactual fairness. Using shorter inputs allowed more consistent and interpretable comparisons between genders, but this inevitably constrains the generalisability of the findings to longer and more complex documents. It is possible that different results could be obtained when longer texts are used, although there is no compelling reason to assume that the gender-specific language generated by models would meaningfully differ solely due to input length.

Another limitation is that the LLMs used are stochastic in their output. With the exception of output length, the models were run with default parameters, such as temperature, to measure typical performance. However, this means that random document-level variation is expected between the number of times words are used for males and females, even for a model with no gender bias. Re-running the code does not yield identical summaries. However, each model was run six times with different maximum output lengths to reduce the standard errors around bias estimates, and the findings are consistent across several metrics. Robustness checks, detailed in the Appendix, consistently yielded the same results. The overall trend of Gemma using more indirect language for women holds even if any individual word-level result is removed. Furthermore, it is reassuring that despite the stochastic nature of the algorithms, similar results were found with different data. As the real administrative data could not be shared, LLMs were used to generate around 400 synthetic case notes, included in this paper’s GitHub repository [53]. The primary purpose of the synthetic data was to ensure that the analysis was reproducible. However, the findings from the synthetic data were found to be consistent with those using the real data. Significant gender-based differences were observed in the summaries generated by the Google Gemma model, with physical and mental health mentioned significantly more in male summaries. Many of the same narrative-type words, such as “text,” “emphasise,” and “describe,” appeared more for women than men, while words relating to needs, such as “require,” “necessitate,” “assistance,” and “old,” appeared more for men. The synthetic data results also show no significant gender-based differences in the Llama 3 model output.

Perhaps a more concerning limitation of the stochastic nature of model output is the difficulty in balancing Type I and Type II errors. With statistical tests performed for thousands of words, some unlikely events are inevitable. Caution was exercised by adjusting the $p$-values (using the Benjamini-Hochberg method), but this means that some words with very small unadjusted $p$-values were rejected. It is possible that some meaningful differences between words on the basis of gender were not considered statistically significant due to this conservatism.

A related point is that meaningful differences will not necessarily generate statistical significance. For instance, in the BART model, the word “unwise” appears 12 times for women and 5 times for men, which is not statistically significant according to a $\chi^2$ test or Fisher’s exact test. However, even a single instance stating that a woman is making unwise decisions, where an otherwise identical man is not described this way, could make a practical difference to a care professional acting upon it.

An additional limitation is that pre-trained sentiment analysis models not trained on health and care data were used. SiEBERT is a transfer learning model built on RoBERTa [37] and fine-tuned on a diverse range of data, including reviews and tweets [36]. Regard is based on BERT and fine-tuned on data designed to evaluate demographic bias [38]. Ideally, domain-specific sentiment analysis models would be used, but such models are not currently available, and constructing them would require subjective judgments about how different conditions or care needs relate to positive or negative sentiment. Future research could benefit from the development of domain-specific models, but the current approach provides meaningful exploration of these differences within the available framework. Despite this limitation, sentiment analysis remains useful for identifying that some measurable difference exists between summaries. The interpretation of these differences becomes clearer through the accompanying analysis of themes and word usage.

Thematic analysis, for example, clearly shows when certain domains (e.g. physical health) are included more often for one gender, as we see in the case of Gemma. Word frequency analysis helps drill down into specific patterns of language. For example, Gemma’s greater use of words such as “text”, “describe”, and “highlight” in summaries for women indicates that it tends to describe the text itself rather than the person receiving care. Together, these methods are specific and interpretable—when they detect a difference, this indicates a meaningful instance of gender bias. However, they may not be sensitive to all types of bias. Subtler forms of framing, tone, or discourse structure may go undetected using these techniques. Pfohl et al. [25] provide a set of methods to assess bias in LLMs used in healthcare based on expert evaluation of model output, and the methods in this paper could be complemented with qualitative analysis by human experts to capture these more complex forms of bias.

Finally, cases relating to gender-specific care, such as mastectomies, and those mentioning domestic violence were removed, as they do not fit easily into the counterfactual fairness framework. However, in some cases, gender is salient and output should legitimately differ based on gender or other protected characteristics [43, 56]. The way language models treat gender-specific circumstances remains an important policy question that should be explored in future work.

## Conclusion

LLM summarisation models are being used in health and care to generate settings and summarise documentation [1–3]. In this study, notable variation in gender-based discrepancies was observed across summarisation LLMs. Llama 3 showed no gender-based differences across any metrics, T5 and BART demonstrated some variation, and the Gemma model exhibited the most significant gender-based disparities. Gemma’s male summaries were generally more negative in sentiment, and certain themes, such as physical health and mental health, were more frequently highlighted for men. The language used by Gemma for men was often more direct, while more euphemistic language was used for women. In the Gemma summaries, women’s health issues appeared less severe than men’s and details of women’s needs were sometimes omitted. Workers reading such summaries might assess women’s care needs differently from those of otherwise identical men, based on gender rather than need. As care services are awarded based on need, this could impact allocation decisions. While gendered language can be appropriate in contexts where gender is relevant, the differences in Gemma’s output suggest that, in many instances, these differences are undesirable.

While this study provides evidence of gender bias in LLM-generated summaries for long-term care, the findings are based on one specific domain and dataset. Further research is needed to assess whether similar patterns arise in other health and care settings, such as hospitals or mental health, where documentation styles and service models may differ. Given the findings in this paper, this makes future research in other health and care contexts where narrative documentation is used an important priority. The methodological framework developed can be applied to any dataset of free-text case records to evaluate bias in model outputs.

As generative models become more widely used for creating documentation, any bias within these models risks becoming part of official records. However, LLMs should not be dismissed as a solution to administrative burden. In this study, there were differences in bias across LLMs. This variation suggests that, if regulators wish to prioritise algorithmic fairness, they should mandate the measurement of bias in LLMs used in long-term care. Practical methods for evaluating gender bias in LLMs have been outlined in this paper, which can be implemented by anyone with access to long-term care data. The code for these evaluations is available on GitHub [53]. It is recommended that these or similar metrics be applied to assess bias across gender, ethnicity, and other legally protected characteristics in LLMs integrated into long-term care systems. By doing so, the benefits of LLMs can be realised while mitigating the risks associated with bias.

## Electronic supplementary material

Below is the link to the electronic supplementary material.

Supplementary Material 1: Three appendices are included: (1) Evaluation of sentiment metrics: establishing which sentiment metrics are appropriate for conducting this analysis. (2) Model diagnostics and robustness checks: verifying the robustness of conclusions using several other methods. (3) Evaluation of themes: full lists of words counted in the frequency of the words appearing in each theme. The code to reproduce this analysis is available with synthetic data in the GitHub repository [53]

## Data Availability

The data that support the findings of this study are individual-level, administrative care records. This is identifiable human data and restrictions apply to the availability of these data, which were used under licence for the current study, and so are not publicly available. It is not possible to share this data publicly as individual privacy could be compromised. Metadata are however available from the authors upon reasonable request. Synthetic data is provided in the GitHub repository [53] so that the methods provided can be reproduced using the code provided. The findings from the synthetic data are consistent with the findings from the real data.

## Abbreviations

AI: Artificial intelligence
ANOVA: Analysis of variance
BART: Bidirectional auto-regressive transformers
BERT: Bidirectional encoder representations from transformers
CAG: Confidentiality advisory group
CI: Confidence interval
EU: European Union
GEE: Generalised estimating equations
GPU: Graphics processing unit
HHS: Health and human services
LLM: Large language model
NHS: National health service
SD: Standard deviation
UK: United Kingdom
US: United States
USD: US Dollars
