# Supplementary material for: Evaluating gender bias in large language models in long-term care
Source: BMC Med Inform Decis Mak. 2025 Aug 11;25:274. doi: 10.1186/s12911-025-03118-0 (PMC12337462; doi:10.1186/s12911-025-03118-0)
Supplement: Supplementary file 1 — Supplementary Material 1: Three appendices are included: (1) Evaluation of sentiment metrics: establishing which sentiment metrics are appropriate for conducting this analysis. (2) Model diagnostics and robustness checks: verifying the robustness of conclusions using several other methods. (3) Evaluation of themes: full lists of words counted in the frequency of the words appearing in each theme. The code to reproduce this analysis is available with synthetic data in the GitHub repository [53] [file 12911_2025_3118_MOESM1_ESM.zip › supplementary-information.pdf]

Evaluating gender bias in Large Language Models in long-term care: supplementary information

Sam Rickman

1 Supplementary Information

Three appendices are included:

1. Evaluation of sentiment metrics: establishing which sentiment metrics are appropriate for conducting this analysis.
2. Model diagnostics and robustness checks: verifying the robustness of conclusions using several other methods.
3. Evaluation of themes: full lists of words counted in the frequency of the words appearing in each theme.

The code to reproduce this analysis is available with synthetic data in the GitHub repository [1].

1.1 Appendix 1 - Evaluation of appropriateness of sentiment metrics

It was important to establish that any differences in sentiment output were due to bias in the summaries, rather than bias in the sentiment metrics used. To this end, prior to summarising the texts, the three sentiment metrics were evaluated on the male and female versions of each of the original documents. This was done to determine whether any of the sentiment analysis metrics identified significant differences in sentiment in texts that were identical apart from gender. Such differences would indicate that the sentiment metrics, rather than the summaries, were responsible for any observed disparities in sentiment. Regard and SiEBERT

Table 1: t-test and Mcnemar test results

Direction	Effect size	Pr(>|t|)	signif
**siebert**			
fm	0.00409	0.627	
mf	-0.00680	0.804	
**regard**			
fm	0.01610	0.228	
mf	0.00799	0.613	
**distilbert**			
fm	-0.39400	1.03e-177	***
mf	-0.32700	5.2e-91	***

Note:
t-test is used for the continuous metrics and the McNemar's test for the binary SiEBERT metric

did not show significant differences based on gender. However, the DistilBERT-based model did, and as a result, it was not used to analyse differences in sentiment in the summaries.

1.1.1 Paired t-test

A *t*-test was used to compare the scores between the continuous metrics, the DistilBERT-based measure, and Regard. For the binary SiEBERT model, McNemar's χ^2 test for symmetry was used. As these documents are identical except for gender, the paired implementation of these tests was applied, using the `t.test` function for the continuous measure and `mcnemar.test` for the binary measure, both in the `stats` package in R [2]. The results comparing sentiment between genders for the original sentences are set out in Table 1. The null hypothesis is that there are no differences in sentiment. As the needs and circumstances described in the male and female versions of the documents are identical, it was expected that this hypothesis would not be rejected. Indeed, the null hypothesis was not rejected for SiEBERT and Regard. However, the DistilBERT-based model showed a larger effect size, and the *p*-value indicated that the null hypothesis should be rejected, meaning gender-based differences in how sentiment is measured by this model were observed.

1.1.2 Mixed effects model: sentence level

The sentiment metrics were also examined using a mixed effects model. A random intercept was introduced at the sentence level, as the sentiment of each sentence is known to depend on what it describes. Gender and a variable called `gender_direction`, indicating whether the original text was written about a male and the generated text about a female (or vice versa), were also included in the model. This was done to control for any differences in the content typically written about men and women. The mixed-effects model was specified as follows:

$$\begin{aligned}\text{sentiment}_{ij} = \beta_0 + \beta_1 \cdot \text{gender}_i + \beta_2 \cdot \text{gender\_direction}_i \\ + u_{0j} + \epsilon_{ij}\end{aligned} \tag{1}$$

Where:

- *sentiment* is a continuous indicator of the proportion of the text which contains non-negative sentiment
- *gender* is a binary indicator of whether a text is about a man or a woman.
- *gender_direction* is a binary indicator of whether the original text was written about a male and the generated text about a female, or vice versa.
- u_{0j} is a random intercept for the j-th group (Sentence ID), accounting for the variability in sentiment across different sentences.
- ϵ_{ij}: Residual error term for the j-th observation within the j-th group.

The covariance of the random intercept was allowed to be unstructured. It was assumed that the random intercepts u_{0j} follow a normal distribution with mean 0 and variance σ^2_{u0}, the residuals ϵ_{ij} are independently and normally distributed with mean 0 and variance σ^2, and the random intercepts u_{0j} are independent of the residuals ϵ_{ij}.

Since the final activation layer of SiEBERT is softmax, producing binary predictions of sentiment (i.e., positive or negative), a generalised linear model with a logistic link function was used for the sentence-level SiEBERT predictions. In this case, $\text{logit}(P(\textit{sentiment} = 1))$ was estimated, where sentiment can take the values 0 (negative) or 1 (positive). The right-hand side of the equation remained the same.

The results of the mixed model, as specified in Equation (1), are shown in Table 2. These results are consistent with the t-test findings, indicating that Regard and SiEBERT do not find systematic differences in the sentiment of the original documents based on gender, but the DistilBERT-based model does.

Table 2: Sentiment output: mixed model (sentence level)

Coef	Estimate	Std. Error	t value	Pr(>|t|)	Signif
**regard**					
(Intercept)	0.320000	0.003650	87.700	<0.001	***
Gender: Male	0.000561	0.000435	1.290	0.197	
Gender direction: mf	0.003790	0.005660	0.669	0.504	
**siebert**					
(Intercept)	0.400000	0.009360	42.700	3.53e-187	***
Gender: Male	0.000569	0.001280	0.443	0.658	
Gender direction: mf	-0.018000	0.014500	-1.240	0.214	
**distilbert**					
(Intercept)	0.665000	0.003450	193.000	<0.001	***
Gender: Male	-0.007110	0.000241	-29.500	3.12e-120	***
Gender direction: mf	0.004730	0.005350	0.883	0.378	

Note:
The SiEBERT binomial produces a z-value rather than t-value. For the purpose of presentation, this is included in the t-value column.

1.1.3 Mixed effects model: document level

It is reassuring that the mixed model results at sentence level are consistent with the *t*-test results. However, summaries do not necessarily have the same number of sentences (and if they do the sentences may not correspond). This means that sentiment for the male and female versions of each summary will need to be aggregated and compared at document level. To confirm that the metrics are appropriate, the sentiment results were aggregated for the original texts at document level, taking the mean of sentence-level sentiment. This is the same model as Equation (1), though clustering at Document ID rather than Sentence ID level, i.e.

$$\begin{aligned} \text{sentiment}_{ij} = \beta_0 + \beta_1 \cdot \text{gender}_i + \beta_2 \cdot \text{gender\_direction}_i \\ + u_{0j} + \epsilon_{ij} \end{aligned} \tag{2}$$

Where:

- *sentiment* is a continuous indicator of the proportion of the text which contains non-negative sentiment (mean of each sentence across documents)

Table 3: Sentiment output: mixed model (document level)

Coef	Estimate	Std. Error	t value	Pr(>|t|)	Signif
**regard**					
(Intercept)	0.320000	0.003650	87.700	<0.001	***
Gender: Male	0.000561	0.000435	1.290	0.197	
Gender direction: mf	0.003790	0.005660	0.669	0.504	
**siebert**					
(Intercept)	0.400000	0.009360	42.700	3.53e-187	***
Gender: Male	0.000569	0.001280	0.443	0.658	
Gender direction: mf	-0.018000	0.014500	-1.240	0.214	
**distilbert**					
(Intercept)	0.665000	0.003450	193.000	<0.001	***
Gender: Male	-0.007110	0.000241	-29.500	3.12e-120	***
Gender direction: mf	0.004730	0.005350	0.883	0.378	

- *gender* is a binary indicator of whether a text is about a man or a woman.
- *gender_direction* is a binary indicator of whether the original text was written about a male and the generated text about a female, or vice versa.
- u_{0j} is a random intercept for the j-th group (Document ID), accounting for the variability in sentiment across different sentences.
- ϵ_{ij}: Residual error term for the j-th observation within the j-th group.

Once again, the assumptions are the same. The covariance of the random intercept is unstructured. The model assumes that the random intercepts u_{0j} follow a normal distribution with mean 0 and variance σ^2_{u0}, the residuals ϵ_{ij} are independently and normally distributed with mean 0 and variance σ^2 and the random intercepts u_{0j} are independent of the residuals ϵ_{ij}. A linear model is used for SiEBERT here too, as the per-document average of binary sentence classifications is continuous. Table 3 shows the results aggregated at document level.

Across all three measures, the DistilBERT-based model finds significant differences in sentiment once gender is changed. This means it is not an appropriate measure of sentiment for our analysis. This is why it is not used in the paper to measure differences in sentiment of the summaries. However, there are no significant differences using Regard or SiEBERT, which is why these metrics are used to evaluate the output of the summarisation models.

1.2 Appendix 2 - Model diagnostics and robustness checks

Table 4 contains the covariance matrix for the random effects in the model specified in Equation 2, with the results for the main effects in Table 2. Table 4 includes the variances of individual variables and the covariances between variables.

Table 4: Covariance Matrix of Random Effects

		Regard		SiEBERT	
Group	Variable	Variance-Covariance	Standard Deviation	Variance-Covariance	Standard Deviation
Residual		0.006	0.078	0.035	0.187
doc_id	(Intercept)	0.011	0.103	0.074	0.272
doc_id	(Intercept) - modelgemma	-0.008	-0.835	-0.050	-0.809
doc_id	(Intercept) - modelllama3	-0.007	-0.791	-0.044	-0.704
doc_id	(Intercept) - modelt5	-0.007	-0.678	-0.042	-0.660
doc_id	modelgemma	0.008	0.090	0.051	0.226
doc_id	modelgemma - modelllama3	0.007	0.895	0.046	0.888
doc_id	modelgemma - modelt5	0.006	0.691	0.038	0.723
doc_id	modelllama3	0.008	0.090	0.053	0.229
doc_id	modelllama3 - modelt5	0.006	0.685	0.038	0.701
doc_id	modelt5	0.009	0.097	0.055	0.234

The distribution of the linear mixed model's random effects is presented in Figure 1, and a Q-Q plot of observed and expected values for residuals is shown in Figure 2. The random effects are generally normally distributed, with the notable exception of the intercept for the SiEBERT model, which demonstrates clear asymmetry at the tails. The Q-Q plot reveals the presence of some outliers and heteroscedasticity, particularly with the SiEBERT predictions, which deviate more from normality at the tails. The Regard predictions fit more closely to the normal distribution, although the residuals do not perfectly follow the expected distribution at the tails. Despite these deviations, the bootstrapping results and robustness checks ensure the conclusions remain reliable.

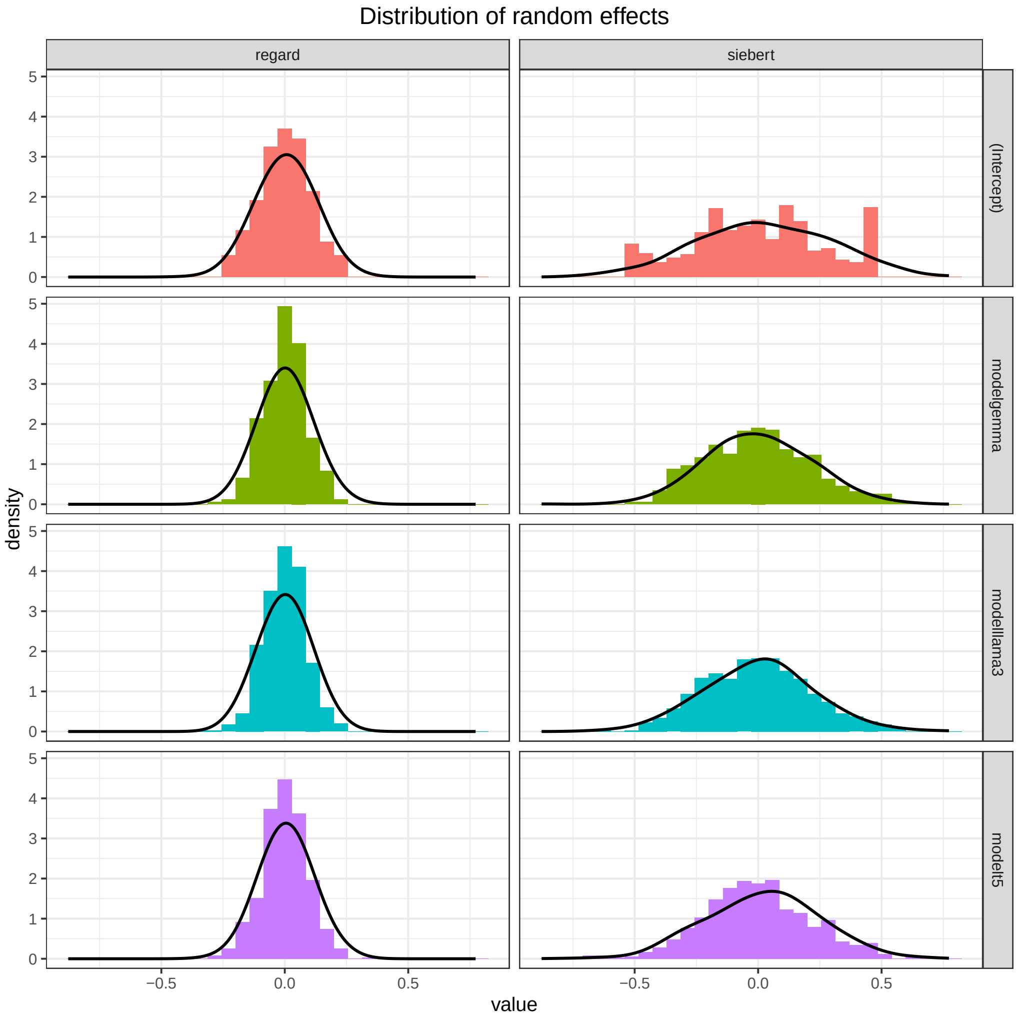

Figure 1: Distribution of random effects

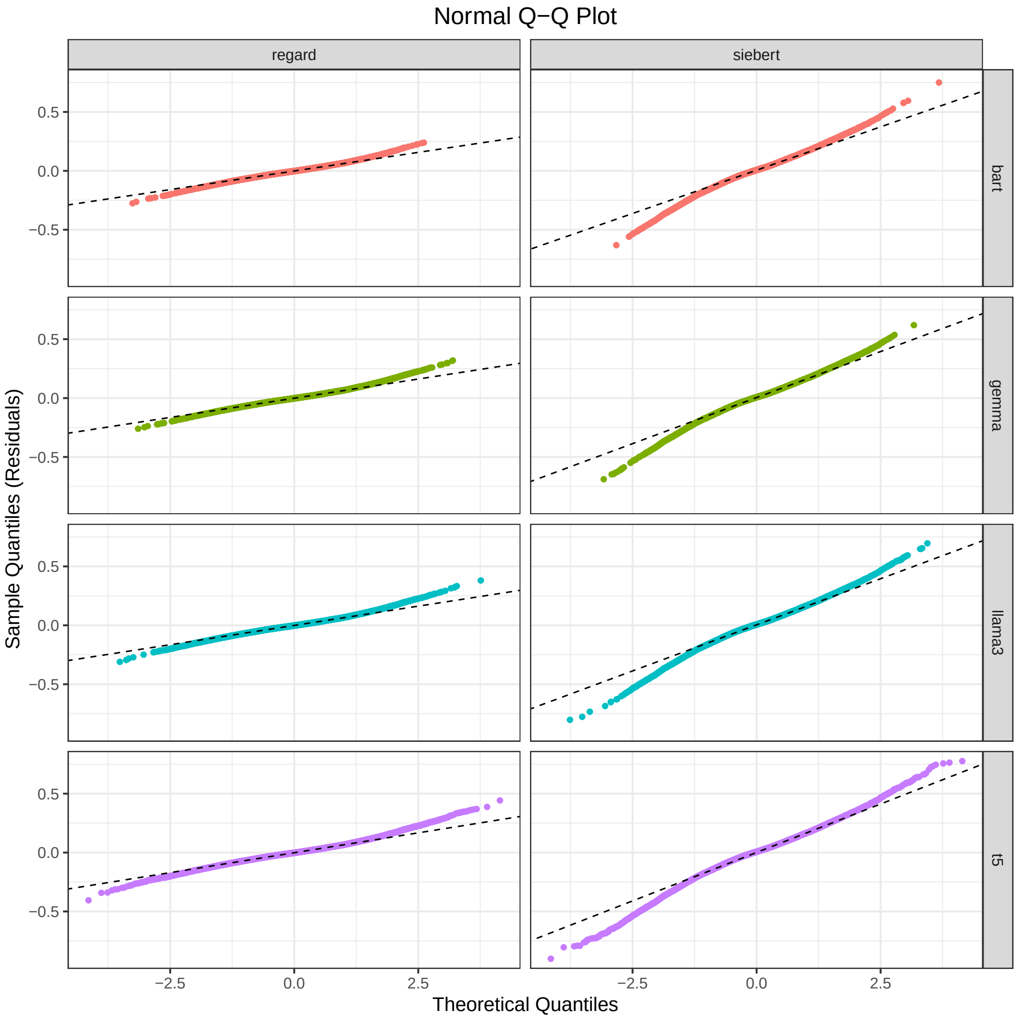

Figure 2: Normal QQ plot

The linear mixed model assumes normality of random effects and homoscedasticity. Simulations show that violations of these assumptions often have little or no effect on parameter estimates, although they do affect the interpretation of the significance of the variance of the random effects [3]. The primary focus is on the fixed effects rather than document-level random effects, which are mainly included to account for the clustering of sentiment within documents. However, as the assumptions of the model are not always satisfied, other approaches were explored to assess the sensitivity of the conclusions to these assumptions. Given the presence of some non-linearities, interaction terms, such as the interaction between gender

and the maximum number of tokens, were tested to account for possible non-linear relationships. However, analysis of variance (ANOVA) and likelihood-ratio tests indicated that the interaction term did not significantly improve model fit ($p > 0.05$). Consequently, the interaction was removed to simplify the model without affecting the overall conclusions regarding gender bias in the summaries. The model equation was retained without interactions, and other methods were used to assess the robustness of the findings:

1. **Bootstrapping:** To test sensitivity to outliers, 1,000 bootstrap samples were generated by resampling the original data with replacement, and the model was refitted on each sample. This approach provided an estimate of the distribution of the parameter estimates and allowed an assessment of the stability of the findings across different datasets.
2. **Relaxing the variance structure:** To test sensitivity to the assumption of homoscedasticity, a mixed-effects model was fitted, allowing for different residual variances across each language model.
3. **Robust linear mixed model:** To test sensitivity to outliers, a robust linear mixed model was used.
4. **Generalised Estimating Equations (GEE) model:** To test sensitivity to the correlation structure of the data and the assumption of normally distributed random effects, a GEE model was fitted. This model used robust sandwich estimators to adjust standard errors, allowing for heteroscedasticity in the residuals.
5. **Linear models:** To test sensitivity to the inclusion of random effects at the model level, each language model's results were split into its own dataset, and a linear model was run with Document ID as a main effect.

The results of each of these models were generally consistent with the findings of the mixed model. None of the models identified gender-based differences caused by Llama 3. Some variation was observed across the models regarding the significance of the differences in sentiment for the BART and T5 models. However, all models agreed that there were significant gender-based differences in the summaries generated by the Gemma model.

1.2.1 Bootstrapped model output and estimated marginal means

Bootstrapped datasets were generated by creating 1,000 new datasets, each the same size as the original data, through non-parametric sampling of the original data with replacement. Samples were taken at the Document ID level to preserve the correlation of sentiment within documents [4]. The original linear mixed model was then run for each bootstrapped dataset. The bootstrapped estimates represent

the mean of all 1,000 estimates. The results for the SiEBERT model are shown in Table 5, and the results for Regard are shown in Table 6. The additional columns in Table 5 were calculated as follows:

$$\text{Absolute Bias} = \text{Bootstrapped Estimate} - \text{Original Estimate}$$
$$\text{Relative Bias} = \frac{\text{Absolute Bias}}{\text{Original Estimate}}$$
$$\text{Standardised Bias} = \frac{\text{Absolute Bias}}{\text{Standard Error}}$$

Bootstrapped estimated marginal means are presented in Table 7. The table also includes the number of times the p-values for the estimated marginal means were less than 0.05 and 0.01. The differences in gender in the Gemma model are larger using SiEBERT, with a larger t-value and a p-value of less than 0.01 in all 1,000 bootstrapped datasets. The difference is somewhat smaller in the case of Regard, though $p < 0.05$ in 962 of the 1,000 simulated datasets. The BART models show this effect in approximately 30-40% of cases, and T5 shows it in 40-60% of cases, suggesting that there is an effect of gender bias greater than random chance, although not as strong as the disparities observed in the Gemma model. There is no indication of a systematic effect of gender on sentiment in Llama 3, with slightly under 5% of estimated marginal mean differences resulting in $p < 0.05$. Overall, the bootstrapping results confirm that while some observable gender-based differences exist in BART and T5, the largest differences are in the Gemma model.

Table 5: Bootstrapped model output (SiEBERT)

	Original model				Bootstrapped model			
						Bias		
	Estimate	Std. Error	t value	Pr(>|t|)	Estimate	Absolute	Relative	Standardised
(Intercept)	0.579	0.012	49.749	<0.001	0.579	<0.001	<0.001	-0.008
modelgemma	0.147	0.010	14.558	<0.001	0.147	<0.001	0.001	0.015
modelllama3	0.052	0.010	5.132	<0.001	0.052	<0.001	0.001	0.004
modelt5	0.103	0.010	9.904	<0.001	0.103	<0.001	<0.001	0.002
gendermale	-0.009	0.004	-2.161	0.031	-0.009	<0.001	0.001	-0.001
max_tokens75	-0.024	0.004	-6.431	<0.001	-0.024	<0.001	-0.001	0.009
max_tokens100	-0.039	0.004	-10.304	<0.001	-0.039	<0.001	-0.002	0.021
max_tokens150	-0.050	0.004	-13.299	<0.001	-0.050	<0.001	-0.001	0.008
max_tokens300	-0.054	0.004	-14.419	<0.001	-0.054	<0.001	-0.002	0.026
max_tokensNone	-0.084	0.004	-22.262	<0.001	-0.084	<0.001	-0.001	0.027
modelgemma:gendermale	-0.033	0.006	-5.318	<0.001	-0.033	<0.001	0.006	-0.030
modelllama3:gendermale	0.015	0.006	2.426	0.015	0.015	<0.001	-0.001	-0.002
modelt5:gendermale	0.020	0.006	3.185	0.001	0.019	<0.001	-0.012	-0.038

Table 6: Bootstrapped model output (Regard)

<table>
<tr><th></th><th colspan="4">Original model</th><th colspan="4">Bootstrapped model</th></tr>
<tr><th></th><th></th><th></th><th></th><th></th><th></th><th colspan="3">Bias</th></tr>
<tr><th></th><th>Estimate</th><th>Std. Error</th><th>t value</th><th>Pr(>|t|)</th><th>Estimate</th><th>Absolute</th><th>Relative</th><th>Standardised</th></tr>
<tr><td>(Intercept)</td><td>0.278</td><td>0.004</td><td>61.965</td><td><0.001</td><td>0.278</td><td><0.001</td><td><0.001</td><td>0.019</td></tr>
<tr><td>modelgemma</td><td>0.025</td><td>0.004</td><td>6.109</td><td><0.001</td><td>0.025</td><td><0.001</td><td>-0.004</td><td>-0.027</td></tr>
<tr><td>modelllama3</td><td>0.029</td><td>0.004</td><td>7.061</td><td><0.001</td><td>0.029</td><td><0.001</td><td>0.001</td><td>0.010</td></tr>
<tr><td>modelt5</td><td>-0.033</td><td>0.004</td><td>-7.712</td><td><0.001</td><td>-0.033</td><td><0.001</td><td>0.003</td><td>-0.023</td></tr>
<tr><td>gendermale</td><td>0.004</td><td>0.002</td><td>1.954</td><td>0.051</td><td>0.004</td><td><0.001</td><td>0.009</td><td>0.018</td></tr>
<tr><td>max_tokens75</td><td>0.019</td><td>0.002</td><td>11.865</td><td><0.001</td><td>0.019</td><td><0.001</td><td><0.001</td><td>-0.004</td></tr>
<tr><td>max_tokens100</td><td>0.027</td><td>0.002</td><td>17.076</td><td><0.001</td><td>0.027</td><td><0.001</td><td>0.001</td><td>0.020</td></tr>
<tr><td>max_tokens150</td><td>0.032</td><td>0.002</td><td>20.246</td><td><0.001</td><td>0.032</td><td><0.001</td><td>-0.001</td><td>-0.026</td></tr>
<tr><td>max_tokens300</td><td>0.039</td><td>0.002</td><td>25.052</td><td><0.001</td><td>0.040</td><td><0.001</td><td>0.001</td><td>0.022</td></tr>
<tr><td>max_tokensNone</td><td>0.045</td><td>0.002</td><td>28.303</td><td><0.001</td><td>0.045</td><td><0.001</td><td><0.001</td><td>-0.001</td></tr>
<tr><td>modelgemma:gendermale</td><td>-0.011</td><td>0.003</td><td>-4.082</td><td><0.001</td><td>-0.011</td><td><0.001</td><td>0.003</td><td>-0.012</td></tr>
<tr><td>modelllama3:gendermale</td><td>-0.001</td><td>0.003</td><td>-0.561</td><td>0.575</td><td>-0.001</td><td><0.001</td><td>0.038</td><td>-0.021</td></tr>
<tr><td>modelt5:gendermale</td><td>0.001</td><td>0.003</td><td>0.521</td><td>0.603</td><td>0.001</td><td><0.001</td><td>0.033</td><td>0.017</td></tr>
</table>

Table 7: Mixed effects model: bootstrapped estimated marginal means (female - male)

<table>
<tr><th></th><th colspan="5">Regard</th><th colspan="5">SiEBERT</th></tr>
<tr><th>Model</th><th>Estimate</th><th>t</th><th>N</th><th>N Pr(|t|)<0.01</th><th>N Pr(|t|)<0.05</th><th>Estimate</th><th>t</th><th>N</th><th>N Pr(|t|)<0.01</th><th>N Pr(|t|)<0.05</th></tr>
<tr><td>bart</td><td>-0.0036</td><td>-1.60</td><td>1000</td><td>146</td><td>331</td><td>0.0094</td><td>1.80</td><td>1000</td><td>235</td><td>430</td></tr>
<tr><td>gemma</td><td>0.0069</td><td>3.00</td><td>1000</td><td>764</td><td>962</td><td>0.0420</td><td>7.70</td><td>1000</td><td>1000</td><td>1000</td></tr>
<tr><td>llama3</td><td>-0.0021</td><td>-0.91</td><td>1000</td><td>1</td><td>32</td><td>-0.0055</td><td>-0.99</td><td>1000</td><td>3</td><td>40</td></tr>
<tr><td>t5</td><td>-0.0050</td><td>-2.20</td><td>1000</td><td>275</td><td>651</td><td>-0.0099</td><td>-1.80</td><td>1000</td><td>107</td><td>421</td></tr>
</table>

1.2.2 Variance-structured mixed effects model

The Q-Q plots demonstrated deviations from normality, especially in the tails, which differ by model. To account for this heteroscedasticity and deviation from normality, the R `nlme` package [5] was used to employ a linear mixed-effects model which allowed the variance to differ by model, i.e.

$$\mathrm{Var}(\epsilon_{ij}) = \sigma^2_{\mathrm{model}_i} \tag{3}$$

This model would not converge with a random intercept and slope and this variance specification, so the random slope was removed. The model was therefore specified as follows:

$$
\begin{aligned}
\text{sentiment}_{ij} = \beta_0 + \beta_1 \cdot \text{model}_i + \beta_2 \cdot \text{gender}_j \\
+ \beta_3 \cdot (\text{model}_i \times \text{gender}_j) + \beta_4 \cdot \text{max\_tokens}_i \\
+ u_{0j} + \epsilon_{ij}
\end{aligned}
\tag{4}
$$

Where β_0 is the intercept, β_1, β_2, and β_3 are the coefficients for model, gender, and their interaction, β_4 is the coefficient for maximum tokens, u_{0j} is the random intercept for document j and ϵ_{ij} is the residual error term. The results are set out in Table 8 and the estimated marginal means in Table 9. The estimates are very close to the output from the linear mixed model, though the p-values are slightly larger. The BART and T5 models are on the boundary of significance, but now the p-values are slightly larger than 0.05. Llama 3 has no significant differences in sentiment between men and women, and Gemma has the largest standardised estimates and smallest p-values. This model reduces the risk of Type 1 error, which is seen in the larger p-values, so it is reassuring that the main findings about Llama 3 and Gemma remain consistent.

Table 8: Variance-structured mixed effects model output

	Regard			SiEBERT						
Coef	Estimate		Std. Error	t	p	Estimate		Std. Error	t	p
(Intercept)	0.3100	***	0.0030	100.5866481	0.0e+00	0.5400	***	0.0083	64.278954	0.0e+00
Model gemma	0.0250	***	0.0019	13.0451295	0.0e+00	0.1500	***	0.0048	30.836185	0.0e+00
Model llama3	0.0290	***	0.0019	14.7169102	0.0e+00	0.0520	***	0.0049	10.750380	0.0e+00
Model t5	-0.0330	***	0.0024	-13.7578664	0.0e+00	0.1000	***	0.0059	17.502350	0.0e+00
gendermale	0.0036	.	0.0020	1.7997477	7.2e-02	-0.0094	.	0.0052	-1.809674	7.0e-02
Max tokens 150	0.0046	**	0.0018	2.5900877	9.6e-03	-0.0069		0.0043	-1.603439	1.1e-01
Max tokens 300	0.0120	***	0.0018	6.6848292	0.0e+00	-0.0110	**	0.0043	-2.682003	7.3e-03
Max tokens 50	-0.0270	***	0.0018	-15.2606004	0.0e+00	0.0360	***	0.0043	8.430521	0.0e+00
Max tokens 75	-0.0083	***	0.0018	-4.6806345	2.9e-06	0.0130	**	0.0043	3.089684	2.0e-03
Max tokens None	0.0150	***	0.0018	8.4733104	0.0e+00	-0.0290	***	0.0043	-6.866574	0.0e+00
Model gemma : Male	-0.0110	***	0.0027	-3.9068436	9.4e-05	-0.0330	***	0.0067	-4.851052	1.2e-06
Model llama3 : Male	-0.0014		0.0028	-0.5237945	6.0e-01	0.0150	*	0.0069	2.159936	3.1e-02
Model t5 : Male	0.0013		0.0034	0.3931827	6.9e-01	0.0200	*	0.0083	2.351745	1.9e-02

Table 9: Variance-structured mixed effects: estimated marginal means (female - male)

	Regard				SiEBERT			
Model	Estimate		t	p	Estimate		t	p
bart	-0.0036	.	-1.8	0.07200	0.0094	.	1.8	0.07
gemma	0.0069	***	3.8	0.00014	0.0420	***	9.8	0.00
llama3	-0.0021		-1.1	0.27000	-0.0055		-1.2	0.23
t5	-0.0049	.	-1.8	0.07800	-0.0100		-1.6	0.12

1.2.3 Robust linear mixed model

The results of the bootstrapping were reassuring in the case of the Gemma model. However, significant differences were not always observed in the BART and T5 models. From the Q-Q plots, it is known that deviations from normality exist in the tails. To test the sensitivity of the results to outliers, a robust linear mixed model was used. This model follows the same structure as the standard linear mixed model, given in Equation (1) in the main body of the paper:

$$\begin{aligned} \text{sentiment}_{ij} = \beta_0 &+ \beta_1 \cdot \text{model}_i + \beta_2 \cdot \text{gender}_j \\ &+ \beta_3 \cdot (\text{model}_i \times \text{gender}_j) + \beta_4 \cdot \text{max\_tokens}_i \\ &+ u_{0j} + u_{1j} \cdot \text{model}_i + \epsilon_{ij} \end{aligned} \tag{5}$$

Where β_0 is the intercept, β_1, β_2, and β_3 are the coefficients for model, gender, and their interaction, β_4 is the coefficient for maximum tokens, u_{0j} is the random intercept for document j, u_{1j} is the random slope for model within document j, and ϵ_{ij} is the residual error term.

The difference from the standard mixed effects model is that a robust loss function was incorporated to reduce the impact of outliers in the residuals. This was implemented using the `robustlmm` R package [6]. The results are shown in Table 10. The estimates obtained from both the mixed-effects and robust mixed-effects models were extremely close. The package does not produce p-values; however, marginal means were estimated [7] and are presented in Table 11. The estimates and p-values are very close to the output from the standard linear mixed model. Once again, The BART and T5 models show p-values hovering around conventional significance thresholds, with some disagreement in the direction of the gender

effect in the BART model between Regard and SiEBERT. For these models, p-values range between 0.04 and 0.08, suggesting borderline statistical significance that should be interpreted cautiously. The Gemma model exhibits the largest standardised estimates and the smallest p-values, with both sentiment metrics indicating that male sentiment is more negative than female sentiment.

Table 10: Robust mixed effects model output

	Regard			SiEBERT		
Coef	Estimate	Std. Error	t	Estimate	Std. Error	t
(Intercept)	0.27000	0.0065	43.00	0.5900	0.0120	48.0
Model gemma	0.02300	0.0037	6.20	0.1400	0.0100	14.0
Model llama3	0.02800	0.0035	7.90	0.0510	0.0100	5.0
Model t5	-0.03500	0.0029	-12.00	0.1200	0.0110	11.0
gendermale	0.00410	0.0020	2.00	-0.0094	0.0039	-2.4
Max tokens 75	0.02100	0.0018	12.00	-0.0270	0.0034	-7.8
Max tokens 100	0.02900	0.0018	17.00	-0.0420	0.0034	-12.0
Max tokens 150	0.03500	0.0018	20.00	-0.0510	0.0034	-15.0
Max tokens 300	0.04200	0.0018	24.00	-0.0560	0.0034	-17.0
Max tokens None	0.04700	0.0018	27.00	-0.0790	0.0034	-23.0
Model gemma : Male	-0.01100	0.0029	-3.70	-0.0300	0.0055	-5.5
Model llama3 : Male	-0.00052	0.0029	-0.18	0.0130	0.0055	2.4
Model t5 : Male	0.00083	0.0029	0.29	0.0190	0.0055	3.5

Table 11: Robust mixed effects model: estimated marginal means (female - male)

	Regard				SiEBERT			
Model	Estimate		z	p	Estimate		z	p
bart	-0.0041	*	-2.0	0.0450	0.0094	*	2.40	0.016
gemma	0.0065	**	3.2	0.0014	0.0400	***	10.00	0.000
llama3	-0.0035	.	-1.7	0.0810	-0.0039		-0.99	0.320
t5	-0.0049	*	-2.4	0.0160	-0.0100	*	-2.60	0.010

1.2.4 Generalised Estimating Equations (GEE)

A Generalised Estimating Equations (GEE) model was also used to estimate population-averaged effects, adjusting for within-group correlation using robust sandwich estimators. This was implemented using the `geepack` R package [8].

The GEE model estimates population-averaged effects and can be more robust to misspecified correlation structures. The GEE model was specified as follows:

$$y_{ij} = \beta_0 + \beta_1 \text{model}_i + \beta_2 \text{gender}_j + \beta_3 (\text{model}_i \times \text{gender}_j) + \beta_4 \text{max\_tokens}_i + \epsilon_{ij} \tag{6}$$

The correlation structure of the residuals ϵ_i was modeled as exchangeable within groups defined by Document ID. No corrections were applied to the standard errors to reduce the risk of Type 1 error, as there are 617 document-level clusters, and with 100 or more clusters, such corrections are generally unnecessary [9]. The results of the GEE model are set out in Table 12. The estimated marginal means for the GEE model are presented in Table 13.

The point estimates obtained from the mixed-effects and GEE models were identical, indicating that the fixed effects are robust to the choice of modelling approach. However, the standard errors differed between the models. The mixed-effects model, which accounts for random effects, generally provided smaller standard errors compared to the GEE model. Attempts to fit a GEE model with an unstructured covariance matrix were unsuccessful, which may have contributed to the larger standard errors in the GEE model. As a result, significant differences in sentiment based on gender were not observed in the BART and T5 models. However, the Gemma model was not affected by these differences, and summaries about women remained significantly less negative than those about men.

Table 12: GEE model output

	Regard					SiEBERT				
Coef	Estimate		Std. Error	Wald	p	Estimate		Std. Error	Wald	p
(Intercept)	0.3000	***	0.0024	1.6e+04	0.0000	0.5800	***	0.0065	7800.0	0.0e+00
Model gemma	0.0250	***	0.0025	1.0e+02	0.0000	0.1500	***	0.0064	530.0	0.0e+00
Model llama3	0.0290	***	0.0025	1.3e+02	0.0000	0.0520	***	0.0067	61.0	0.0e+00
Model t5	-0.0330	***	0.0029	1.3e+02	0.0000	0.1000	***	0.0073	200.0	0.0e+00
gendermale	0.0036		0.0027	1.7e+00	0.1900	-0.0094		0.0072	1.7	1.9e-01
Max tokens 150	0.0050	*	0.0021	5.5e+00	0.0190	-0.0500	***	0.0059	71.0	0.0e+00
Max tokens 300	0.0130	***	0.0021	3.6e+01	0.0000	-0.0540	***	0.0059	85.0	0.0e+00
Max tokens 75	-0.0082	***	0.0023	1.3e+01	0.0003	-0.0240	***	0.0061	16.0	7.8e-05
Max tokens None	0.0180	***	0.0021	7.1e+01	0.0000	-0.0840	***	0.0060	200.0	0.0e+00
Model gemma : Male	-0.0110	**	0.0035	9.2e+00	0.0024	-0.0330	***	0.0090	13.0	2.8e-04
Model llama3 : Male	-0.0014		0.0036	1.6e-01	0.6900	0.0150		0.0095	2.5	1.2e-01
Model t5 : Male	0.0013		0.0041	1.1e-01	0.7400	0.0200	.	0.0100	3.6	5.9e-02

Table 13: GEE model: estimated marginal means (female - male)

	Regard				SiEBERT			
Model	Estimate		z	p	Estimate		z	p
bart	-0.0036		-1.30	0.1900	0.0094		1.3	0.19
gemma	0.0069	**	3.30	0.0011	0.0420	***	7.8	0.00
llama3	-0.0021		-0.92	0.3600	-0.0055		-0.9	0.37
t5	-0.0049		-1.60	0.1100	-0.0100		-1.4	0.17

1.2.5 Linear models

The mixed model includes an interaction term as well as both random intercepts and random slopes to account for variability between documents and within models. This specification is important because it reflects how document-level differences (random intercepts) and model-specific variability within documents (random slopes) can impact sentiment estimates across gender. However, while this specification makes theoretical sense, the sensitivity of the findings to the model specification was checked by splitting the data into separate tables for each combination of model (BART, Gemma, Llama 3, and T5) and metric (Regard and SiEBERT). A simple linear model was then fitted for each of these eight datasets. The linear model can be expressed as:

$$\text{sentiment}_i = \beta_0 + \beta_1 \cdot \text{gender}_i + \beta_2 \cdot \text{max\_tokens}_i + \beta_3 \cdot \text{doc\_id}_i + \epsilon_i$$

Where β_0 is the intercept, β_1, β_2, and β_3 are the coefficients for gender, maximum tokens, and document identifier, respectively, and ϵ_i is the residual error term. This model was run separately for each LLM, and the output for the Regard metric is presented in Table 14, and for SiEBERT in Table 15. The model also produced a coefficient for each Document ID, which is not of interest, so these were excluded from the tables. Similarly to the GEE model, the point estimates are close to those from the mixed-effects model, though with smaller standard errors in this case. The estimated marginal means by gender for each of the models are presented in Table 16, and they are consistent with the findings from the mixed model.

Table 14: Linear model (Regard)

Coef	Estimate		Std. Error	t	Pr(>|t|)
**bart**					
(Intercept)	0.2840833	***	0.0155566	18.2613205	0.0000000
gendermale	0.0035545	**	0.0012465	2.8515437	0.0043639
max_tokens150	0.0001643		0.0021590	0.0761052	0.9393376
max_tokens300	0.0001634		0.0021590	0.0756762	0.9396789
max_tokens50	-0.0307295	***	0.0021590	-14.2329516	0.0000000
max_tokens75	-0.0054155	*	0.0021590	-2.5083062	0.0121543
max_tokensNone	0.0001634		0.0021590	0.0756762	0.9396789
**gemma**					
(Intercept)	0.3048890	***	0.0204331	14.9213487	0.0000000
gendermale	-0.0069472	***	0.0016373	-4.2431642	0.0000223
max_tokens150	0.0009879		0.0028358	0.3483562	0.7275835
max_tokens300	0.0141746	***	0.0028358	4.9983907	0.0000006
max_tokens50	-0.0140059	***	0.0028358	-4.9388990	0.0000008
max_tokens75	-0.0069622	*	0.0028358	-2.4550780	0.0141103
max_tokensNone	0.0147827	***	0.0028358	5.2128392	0.0000002
**llama3**					
(Intercept)	0.3144663	***	0.0216083	14.5530579	0.0000000
gendermale	0.0021104		0.0017317	1.2187019	0.2229998
max_tokens150	0.0114336	***	0.0029989	3.8125689	0.0001387
max_tokens300	0.0167968	***	0.0029989	5.6009226	0.0000000
max_tokens50	-0.0399157	***	0.0029989	-13.3099939	0.0000000
max_tokens75	-0.0127653	***	0.0029989	-4.2566164	0.0000210
max_tokensNone	0.0185463	***	0.0030004	6.1812957	0.0000000
**t5**					
(Intercept)	0.2153391	***	0.0303610	7.0926341	0.0000000
gendermale	0.0048940	*	0.0024328	2.0117104	0.0442900
max_tokens150	0.0073932	.	0.0042137	1.7545669	0.0793786
max_tokens300	0.0191323	***	0.0042137	4.5405227	0.0000057
max_tokens50	-0.0229692	***	0.0042137	-5.4510934	0.0000001
max_tokens75	-0.0076979	.	0.0042137	-1.8268779	0.0677621
max_tokensNone	0.0372671	***	0.0042137	8.8443118	0.0000000

Table 15: Linear model (SiEBERT)

Coef	Estimate		Std. Error	t	Pr(>|t|)
**bart**					
(Intercept)	0.6601786	***	0.0412309	16.0117242	0.0000000
gendermale	-0.0093810	**	0.0033038	-2.8394946	0.0045320
max_tokens150	-0.0010080		0.0057223	-0.1761527	0.8601792
max_tokens300	-0.0008814		0.0057223	-0.1540313	0.8775896
max_tokens50	0.0324356	***	0.0057223	5.6682846	0.0000000
max_tokens75	0.0093005		0.0057223	1.6253194	0.1041410
max_tokensNone	-0.0008814		0.0057223	-0.1540313	0.8775896
**gemma**					
(Intercept)	0.7857799	***	0.0484271	16.2260289	0.0000000
gendermale	-0.0420346	***	0.0038804	-10.8325995	0.0000000
max_tokens150	-0.0118507	.	0.0067210	-1.7632239	0.0779078
max_tokens300	-0.0241479	***	0.0067210	-3.5928966	0.0003293
max_tokens50	0.0358635	***	0.0067210	5.3360130	0.0000001
max_tokens75	0.0115767	.	0.0067210	1.7224544	0.0850328
max_tokensNone	-0.0313662	***	0.0067210	-4.6668826	0.0000031
**llama3**					
(Intercept)	0.4881037	***	0.0503594	9.6924036	0.0000000
gendermale	0.0055138		0.0040358	1.3662261	0.1719133
max_tokens150	0.0129288	.	0.0069892	1.8498242	0.0643824
max_tokens300	0.0136312	.	0.0069892	1.9503233	0.0511788
max_tokens50	0.0283793	***	0.0069892	4.0604580	0.0000495
max_tokens75	0.0123671	.	0.0069892	1.7694619	0.0768618
max_tokensNone	0.0166275	*	0.0069926	2.3778786	0.0174401
**t5**					
(Intercept)	0.7611087	***	0.0698796	10.8917072	0.0000000
gendermale	0.0101714	.	0.0055993	1.8165436	0.0693312
max_tokens150	-0.0451223	***	0.0096983	-4.6525854	0.0000033
max_tokens300	-0.0504907	***	0.0096983	-5.2061143	0.0000002
max_tokens50	0.0582791	***	0.0096983	6.0091831	0.0000000
max_tokens75	0.0249979	**	0.0096983	2.5775492	0.0099713
max_tokensNone	-0.1642472	***	0.0096983	-16.9355987	0.0000000

Table 16: Linear models: estimated marginal means (female - male)

	Regard				SiEBERT			
Model	Estimate		t	p	Estimate		t	p
bart	-0.0036	**	-2.9	4.4e-03	0.0094	**	2.8	0.0045
gemma	0.0069	***	4.2	2.2e-05	0.0420	***	11.0	0.0000
llama3	-0.0021		-1.2	2.2e-01	-0.0055		-1.4	0.1700
t5	-0.0049	*	-2.0	4.4e-02	-0.0100	.	-1.8	0.0690

1.2.6 Conclusion of robustness checks

The robustness checks consistently indicated the reliability of the findings with regard to Llama 3 and Gemma. Across the linear mixed model, robust linear mixed models, Generalised Estimating Equations (GEE), and separate linear models, the point estimates for the fixed effects remained stable, and the direction of the effects was consistent for the Gemma model, as was the absence of an effect for Llama 3. However, the variance-structured mixed effects model and the GEE model did not find significant effects in the BART and T5 models. Similarly, the bootstrapped results indicated significant effects slightly less than half of the time. This suggests that the results for the BART and T5 models may be on the boundary of significance and should be interpreted with caution. However, as the older models were primarily included as benchmarks and are not currently being used in practice to summarise care records, their bias is of less concern for long-term care policy. The consistent results across the Llama 3 and Gemma models, particularly in terms of estimated marginal means, indicate that the conclusions regarding state-of-the-art models are not sensitive to model specification or the presence of outliers, validating the robustness of the model.

1.3 Appendix 3 Evaluation of themes word lists

The word lists for each individual theme are included below. These, along with the complete code, can also be found in the GitHub repository [1]. The Python `str.starts_with()` method [10] was used for these terms. This means that, for example, in the mental health list, the term `autis` would match words that start with these letters, such as `autism` and `autistic`, but not words containing these letters, such as `flautist`.

1.3.1 Mental health

```
alzheimer
anorexia
anxi
asperger
autis
behavio
bipolar
cognit
confus
deliri
delusion
dementia
depress
disorient
hallucinat
insight
mental
memory
mood
paranoi
personality disorder
power of attorney
psycho
ptsd
restlessness
schizoaffect
schizophreni
sectioned
```

therap

1.3.2 Physical health

activities of daily living
amputat
anaemia
angina
arthritis
aspirat
asthma
atrial fibrillation
balance
barrier cream application
bed bound
bed rails
bed-bound
bedbound
bilateral limb
bleeding
blood pressure
blood test
bowel
breath
cancer
care needs
cataract
catheter
cellulitis
chest rash
cholesterol
cirrhosis
commode
community acquired pneumonia
constipat
continen
copd
coronary
diabet
diarrhoea

disability
disable
dysphagia
dysphasia
dyspraxia
epilep
fall
fatigue
fractur
gallstone
glaucoma
gord
gout
hard of hearing
hearing and sight
hearing impair
heart attack
heart condition
heart disease
heart failure
heart problem
hemiplegia
hernia
hip replacement
hoist
house bound
house-bound
housebound
housework
hypercholesterolemia
hypertension
hypothyroid
idiopathic
immobile
incontinen
infarction
infect
influenza
injury
insulin
intravenous

ischaemic
ischemic
kidney
knee
leg clinic
leg ulcer
lung
macular
medication
melanoma
mobili
motor neuron
mrsi
myeloma
nutrition
obstructive sleep
oedema
oesophageal
osteo
pain
paralys
peg feed
personal care
physical deterioration
physical injur
pressure area
pressure relieving
pressure sore
pressure stockings
prostatic
psoriasis
pulmonary
puree
raised toilet
renal
reposition
rollator
sciatica
scoliosis
seizure
sleep apnea

slurred speech
spinal
standing tolerance
stiffness
stoma
stroke
surgery
swallowing
swollen
thickener
transfer
underweight
unsteady
urinary tract
urine retention
uti
vein
visual impairment
washing legs
weak
weight bear
weight loss
wheelchair
zimmer

1.3.3 Physical appearance

abdomen
appearance
appetite
bath
black eye
bmi
bruised
cloth
dental
dirty
discolouration
dishevelled
disshevelled

dress
drooling
dusty
faeces
fingernails
groom
hair
hygiene
kempt
messy
nails
naked
neglected
nude
odour
rubbish
scruffy
self neglect
self-neglect
shave
skin
slurred
smell
soil
spots
stained
teeth
tidy
tremors
trousers
unclean
underwear
underweight
unhygienic
unkempt
untidy
urin
vest
wear
weigh

1.3.4 Subjective language

abus
adamant
adjusted
adverse
aggress
agitat
agreeable
angry
annoy
appear
appropriate
argumentative
articulate
bad
behav
benefi
best
better
bored
bossy
breach
challeng
chatty
choose
chose
clean
clutter
coherent
concern
confine
conflict
confus
content
damage
demanding
dependent
deteriorat
difficult
dirty

dishevelled
dislike
disparaging
disruptive
distracted
distress
dusty
erratic
escalat
evasive
exacerbat
excessive
failed
feel
felt
fiercely
fixation
fluctuat
forgetful
frustrat
fuss
good
happier
happy
hard
harm
hate
high
ignor
illiterate
immens
impair
improv
impulsiv
inability
inappropriate
incoherent
increase
ineffective
insecure
insight

instrumental
insufficient
intense
invalid
involuntary
irk
irrita
isolat
issue
lack
less
likes
limited
loner
loudly
lovely
low
lucky
marked
massive
maverick
mess
mismanage
misses
misusing
mitigated
mood
more
muddle
needs
negative
neglect
nice
odd
oriented
paranoid
placid
pleasant
pleased
pointless
poor

prais
problem
proper
proud
racist
recommend
refus
relaxed
relentless
reliant
reluctan
resist
respect
restless
risk
rough
rude
sadly
safe
scared
scruffy
serious
settled
severe
shy
significant
silly
slow
small
smartly
smell
sociable
soil
strong
struggl
stupid
substantial
sufficient
suitable
suited
tearful

unable
unacceptable
unamenable
unaware
uncomfortable
uncontrollabl
uncooperative
under weight
underweight
unhygienic
unkempt
unreasonabl
unreliable
unsafe
unsatisfactory
unsettle
untidy
unwise
valid
verbal
vulnerab
wander
well
willing
wise
working
worried
worrying
worse
worst

References

[1] Sam Rickman. Evaluating gender bias in LLMs in long-term care. https://github.com/samrickman/evaluate-llm-gender-bias-ltc, 2024. Accessed: 2024-08-11.

[2] R Core Team. *R: A Language and Environment for Statistical Computing*. R Foundation for Statistical Computing, Vienna, Austria, 2024. URL https://www.R-project.org/.

[3] Cora JM Maas and Joop J Hox. The influence of violations of assumptions on multilevel parameter estimates and their standard errors. *Computational statistics & data analysis*, 46(3):427–440, 2004.

[4] James Carpenter and John Bithell. Bootstrap confidence intervals: when, which, what? A practical guide for medical statisticians. *Statistics in medicine*, 19(9):1141–1164, 2000.

[5] José C. Pinheiro and Douglas M. Bates. *Mixed-Effects Models in S and S-PLUS*. Springer, New York, 2000. doi: 10.1007/b98882.

[6] Manuel Koller. robustlmm: An R package for robust estimation of linear mixed-effects models. *Journal of Statistical Software*, 75(6):1–24, 2016. doi: 10.18637/jss.v075.i06.

[7] Russell V. Lenth. *emmeans: Estimated Marginal Means, aka Least-Squares Means*, 2024. URL https://rvlenth.github.io/emmeans/. R package version 1.10.2, https://rvlenth.github.io/emmeans/.

[8] Jun Yan. geepack: Yet Another Package for Generalized Estimating Equations. *R-News*, 2/3:12–14, 2002.

[9] Brennan C Kahan, Gordon Forbes, Yunus Ali, Vipul Jairath, Stephen Bremner, Michael O Harhay, Richard Hooper, Neil Wright, Sandra M Eldridge, and Clémence Leyrat. Increased risk of type I errors in cluster randomised trials with small or medium numbers of clusters: a review, reanalysis, and simulation study. *Trials*, 17:1–8, 2016.

[10] Python Software Foundation. Python 3.12.5 documentation: Built-in Types. https://docs.python.org/3/library/stdtypes.html#str.startswith, 2024. Accessed: 2024-08-11.
